# Sensitive Detection of KRAS Mutations by Clustered Regularly Interspaced Short Palindromic Repeats

**DOI:** 10.3390/diagnostics11010125

**Published:** 2021-01-15

**Authors:** Huifen Zhou, Jen-Hui Tsou, Qixin Leng, Feng Jiang

**Affiliations:** Department of Pathology, University of Maryland School of Medicine, 10 S. Pine St., Baltimore, MD 21201, USA; Huifen.Zhou@som.umaryland.edu (H.Z.); JTsou@som.umaryland.edu (J.-H.T.); QLeng@som.umaryland.edu (Q.L.)

**Keywords:** CRISPR, tissues, biomarkers, cancer, diagnosis

## Abstract

Kirsten rat sarcoma viral oncogene (KRAS) is the isoform most frequently mutated in human tumors. Testing for activating KRAS mutations has important implications for diagnosis and the personalized medicine of cancers. The current techniques for detecting KRAS mutations have moderate sensitivity. The emerging clustered regularly interspaced short palindromic repeats (CRISPR) system shows great promise in the detection of nucleic acids and is revolutionizing medical diagnostics. This study aimed to develop CRISPR–Cas12a as a sensitive test to detect KRAS mutations. Serially diluted DNA samples containing KRAS mutations are subjected to CRISPR–Cas12a and polymerase chain reaction (PCR). CRISPR–Cas12a and PCR can specifically detect 0.01% and 0.1% mutant KRAS DNA in the presence of wild-type KRAS DNA, respectively. Twenty pairs of lung tumor and noncancerous lung tissues are tested by CRISPR–Cas12a, PCR, and direct sequencing. CRISPR–Cas12a could identify the G12C mutation in five of 20 tumor tissues, while both PCR and direct sequencing discovered the KRAS mutation in three of the five tumor tissues. Furthermore, the results of CRISPR–Cas12a for testing the mutation could be directly and immediately visualized by a UV light illuminator. Altogether, CRISPR–Cas12a has a higher sensitivity for the detection of KRAS mutations compared with PCR and sequencing analysis, and thus has diagnostic and therapeutic implications. Nevertheless, the technique needs to be validated for its clinical significance in a large and prospective study.

## 1. Introduction

Single nucleotide mutations of Kirsten rat sarcoma viral oncogene (KRAS) frequently occur in and near codons of KRAS and drive the transformation of normal cells into cancerous cells [1]. The activating KRAS mutations are found in numerous malignancies with a frequency of 90% in pancreatic cancer, 35% in lung cancer, and 30%–60% in colon cancer [1,2]. G12 mutations comprise 83% of all KRAS mutations, followed by G13 mutations (14%) and Q61 mutations (2%) [1,2]. Therefore, testing for the activating KRAS mutations, particularly G12 mutations, could be used to diagnose primary tumors [1,2]. Furthermore, since cancer patients with KRAS mutations do not benefit from anti-epidermal growth factor receptor (EGFR) monoclonal antibodies, KRAS mutation testing can guide anti-EGFR therapy selection [3]. At present, several techniques including polymerase chain reaction (PCR) and sequencing analyses are used to detect KRAS mutations in clinical specimens. However, the overall sensitivity of the platforms is not enough for the detection of KRAS mutations [4]. Therefore, a highly sensitive assay for detecting KRAS mutations is urgently needed in clinical settings.

Clustered regularly interspaced short palindromic repeats (CRISPR) are a family of DNA sequences found within the genomes of prokaryotic organisms [5,6,7]. The CRISPR-associated (Cas) immune system has been applied in molecular biology to target and cleave specific nucleic acid sequences, which is commonly used in gene editing [8]. Furthermore, upon binding to target double-stranded (ds) DNA, Cas12a proteins can be activated and unleash the nonspecific endoribonuclease activity to degrade single-stranded (SS) DNA. Therefore, CRISPR shows great promise in the detection of nucleic acids and is revolutionizing medical diagnostics [5,6,7,8]. For instance, we recently demonstrated that CRISPR–Cas12a could sensitively detect nuclei acids of exogenous viruses, such as human papillomavirus, in plasma without requiring ancillary machineries [9]. We have also demonstrated that CRISPR–Cas12a can discover endogenous DNA mutations of tumor-related genes in plasma [10]. In this study, we investigated whether CRISPR–Cas12a could sensitively detect KRAS mutations.

## 2. Materials and Methods

**Cell culture.** Two lung cancer cell lines (H1792 and A549), a human pancreatic cancer cell line (PANC-1), and an immortalized human lung epithelial cell line (BEAS-2B) were obtained from the American Type Culture Collection (ATCC) (ATCC, Manassas, VA, USA) and cultured according to the manufacturer’s instructions. Genomic DNA of H1792 cancer cell line is homozygous for KRAS G12C (34G > T) mutation. Genomic DNA of A549 cancer cell line is homozygous for G12S (34G > A) mutation. Genomic DNA of PANC-1 cancer cell line is homozygous for KRAS G12D (35G > A) mutation. BEAS-2B cells have wild-type KRAS.

**Plasmids.** Plasmids of KRAS mutation G12C and G12S (#83167 and #83144) in Escherichia coli DH5α were obtained from Addgene (Addgene, Watertown, MA, USA) and grown in LB medium with 50 µg/mL Streptomycin in a shaking incubator. Plasmid DNA was isolated by using our previously developed protocols [9,10]. DNA concentration was measured by using the Quantifiler Human DNA Quantification kit (Applied Biosystems, Foster City, CA, USA). Plasmid DNA was serially diluted in the DNA of peripheral blood mononuclear cells (PBMCs) of a healthy donor to determine the analytic performance of molecular tests.

**Clinical specimens.** With a study protocol approved by the Institutional Review Board of the University of Maryland Baltimore, we obtained 20 frozen lung tumor tissues and the matched noncancerous lung tissues from a tissue bank of the University Medical Center. Representative sections from tissues were stained with H&E and reviewed by two pathologists to secure that the tumor tissues had more than 85% cancer cells. The clinical diagnosis of lung cancer was made using histopathologic examinations of specimens obtained by CT-guided transthoracic needle biopsy, transbronchial biopsy, video-assisted thoracoscopic surgery (VATS), or surgical resection. The surgical pathologic staging was determined according to the TNM classification of the International Union Against Cancer with the eighth American Joint Committee on Cancer and the International Staging System for Lung Cancer. Histopathological classification was determined according to the World Health Organization classification.

**DNA extraction.** We extracted DNA using the DNeasy kit (Qiagen, Valencia, CA, USA) as described in our previous publications [9,10,11,12,13,14]. We eluted DNA with 50 μL of elution buffer (10 mmol/L Tris-Cl, pH 8.5) (Sigma-Aldrich Corporation). DNA was quantified using the Quantifiler Human DNA Quantification kit (Applied Biosystems).

**Polymerase chain reaction (PCR)**. We used a PCR-based AmoyDx KRAS mutation detection kit (Xiamen, China) for testing mutations according to the manufacture’s instruction. Briefly, each reaction was prepared in a total volume of 50 µL containing 1X TaqMan™ Universal PCR Master Mix, no AmpErase™ UNG (ThermoFisher), 5 µL DNA solution, and KRAS mutation G12C and G12S specific primer pairs. PCR was performed using the Bio-Rad CFX96 Real-Time System (Bio-Rad). The PCR temperature cycling conditions were as follows: an initial incubation at 95 °C for 2 min followed by 38 to 40 cycles of alternating 95 °C for 30 s, 50.8 °C for 30 s and 72 °C for 30 s. A further incubation at 72 °C for 5 min was carried out to complete the extension step. The cut-off levels to determine the presence of the KRAS mutations were provided by the manufacturer.

**Detection of KRAS mutations by CRISPR–Cas12a.** We designed crRNAs (gRNAs) that specifically targeted three KRAS mutation sites (G12C, G12S, and G12D), respectively. Sequences of the crRNAs and reporter substrates are listed in Table 1.

**Figure 1:**
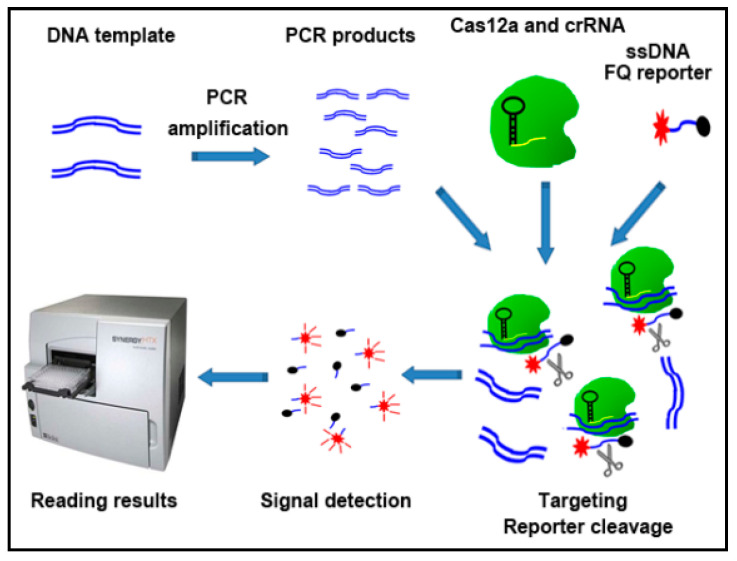
Overview of the CRISPR–Cas12a system to detect Kirsten rat sarcoma viral oncogene (KRAS) mutations. DNA was processed simply for PCR amplification. The PCR product was a mixture of LbaCas12a, crRNA, and ssDNA FQ reporter. Reaction was performed at room temperature on a plate reader for fluorescent readout.

As shown in Figure 1, the CRISPR–Cas12a detection system comprised three steps.

First, DNA template was simply amplified by using PCR. The sequences of the primers for PCR are shown in Supplemental Table S1. PCR amplifications were carried out with a denaturing step at 94 °C for 3 min, 10 s at 94 °C, 30 s at 60 °C for 39 cycles. Second, 250 nM LbaCas12a (Addgene) was pre-assembled with 500 nM crRNAs (Integrated DNA Technologies, IDT, Coralville, IA, USA) and 500 nM of ssDNA-FQ reporter (IDT) in 1X binding buffer (20 mM Tris-HCl, pH 7.5, 100 mM KCl, 5 mM MgCl2, 1 mM DTT, 5% glycerol, 50 µg/mL heparin). The pre-assembled mixture was added to 5 µL of DNA solution (10 ng/µL) in total 20 µL volumes. Third, the results were read on a fluorescence plate reader (Biotek^®^ Synergy™ H1, Winooski, VT, USA) (Promega Corporation, Madison, WI, USA) with emission filters (λex: 485 nM; λem: 535 nM) for fluorescent readout.

**Visual detection of CRISPR–Cas12a activity using a UV light illuminator.** CRISPR–Cas12a reaction was carried out as described above. The visual detection was based on the reaction solution’s fluorescence signal, in which, we captured tubes’ images in the Bio-Rad ChemiDoc™ MP Imaging system (Bio-Rad Laboratories, Hercules, CA, USA) with its built-in UV channel.

**Direct sequencing analysis.** We used one pair of primers to amplify KRAS exon 2. Sequences of the primers are shown in Supplemental Table S2. We carried out PCR with a denaturing step at 94 °C for 3 min, 10 s at 94 °C, 30 s at 60 °C for 39 cycles. The PCR product was visualized on 1.0% agarose gel and purified by Qiagen Gel Extraction kit. Sequencing was performed in the 3730 XL DNA Analyzer (Thermos Fisher Scientific, Waltham, MA, USA) at the University of Maryland Core Facility according to a standard protocol [2].

**Statistical analysis.** We used Fisher’s exact test and Mann–Whitney U-test to analyze the variables. All statistical tests were two sided. A *p*-value ≤ 0.05 was statistically significant. We reported the results as two-sided *p*-values with 95% confidence intervals (95% CI). Statistical analysis was performed with Prism (Graphpad Software, San Diego, CA, USA).

## 3. Results

### 3.1. CRISPR–Cas12a Can Detect KRAS Mutations

**Figure 2:**
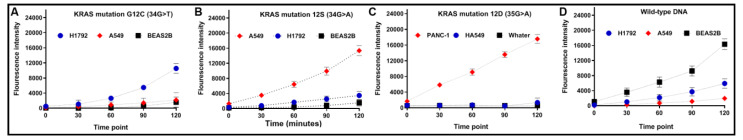
Time courses of fluorescence detection of CRISPR–Cas12a with specific crRNAs in cell lines: (**A**) fluorescence intensity of CRISPR–Cas12a with specific crRNA targeting KRAS 12C significantly raised with an increase in reaction time in DNA of H1792 cells that had G12C mutation (*p* = 0.011); (**B**) fluorescence intensity of CRISPR–Cas12a with specific crRNA targeting KRAS 12S significantly elevated with the reaction time in A549 cells that had G12S mutation (*p* = 0.003); (**C**) fluorescence intensity of CRISPR–Cas12a with specific crRNA targeting KRAS 12D significantly elevated with the reaction time in PANC-1 cells that possessed G12D mutation (*p* = 0.001); and (**D**) fluorescence intensity of CRISPR–Cas12a with specific crRNA targeting wild-type KRAS increased with the reaction time in BEAS2B cells that had wild-type KRAS (*p* = 0.008).

Fluorescence intensity of CRISPR–Cas12a with specific crRNAs against G12S, G12C, or G12D mutation augmented with the reaction time in DNA of lung cancer cell lines that had the mutations (Figure 2A–C). However, CRISPR–Cas12a with the specific crRNAs for the mutations did not lead to increased fluorescence values in wild-type KRAS DNA samples. Conversely, the fluorescence intensity of CRISPR–Cas12a with crRNAs specific to wild-type KRAS increased with the reaction time in the wild-type KRAS DNA; however, this did not cause a change in the fluorescence intensity in DNA of the lung cancer cell lines that had the KRAS mutations (Figure 2D). The results suggested the feasibility of CRISPR–Cas12a for KRAS mutation detection.

### 3.2. CRISPR–Cas12a Has a Higher Sensitivity for Detecting KRAS Mutations Compared with PCR Assay

**Figure 3:**
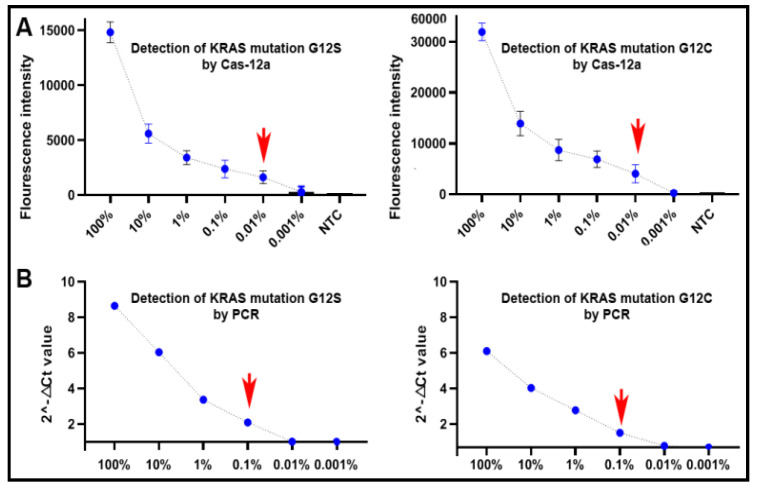
The dynamic ranges and sensitivities of CRISPR–Cas12a and PCR for the detection of KRAS mutations in serially diluted samples with different proportions of mutant KRAS DNA and wild-type KRAS DNA in the DNA of peripheral blood mononuclear cells (PBMCs) of a healthy donor: (**A**) CRISPR–Cas12a had a dynamic range of five orders of magnitude from 0.01% to 100% with a limit of detection (LOD) of 0.01% in the detection of the mutations; (**B**) PCR had a dynamic range of four orders of magnitude ranging from 0.1% to 100% with an LOD of 0.1%. The red arrow indicates the LOD of the tests. NTC, negative template control.

Genomic DNA of plasmids containing an aKRAS G12C or G12S mutation, respectively, was serially (10-fold) diluted into the DNA of PBMCs of a healthy donor by the following mutation allele frequencies of 0.001%, 0.01%, 0.1%, 1%, 10% and 100%. The serially diluted samples were subjected to CRISPR–Cas12a and PCR analyses for mutation detection. As shown in Figure 3A, CRISPR–Cas12a could detect the KRAS mutations over the range of 100% to 0.01%, implying that it had a limit of detection (LOD) of a mutation presence at 0.01% (27 copies of mutant DNA/µL) in a background of wild-type genomic DNA. PCR had a LOD of 0.1% (270 copies/µL) mutated DNA in wild-type DNA (Figure 3B). As a result, CRISPR–Cas12a had a higher sensitivity (10-fold) compared to PCR for detecting the KRAS mutations (*p* < 0.05).

### 3.3. CRISPR–Cas12a Can Specifically Detect KRAS Mutations

**Figure 4:**
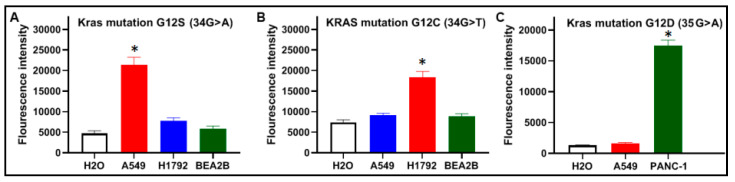
Specificity of CRISPR–Cas12a with crRNAs for the detection of KRA mutations: (**A**) CRISPR–Cas12a with crRNA targeting KRAS 12S identified the mutation only in the DNA of A549 cells that carry KRAS 12S; (**B**) CRISPR–Cas12a with crRNA targeting KRAS 12C detect the mutation only in the DNA of H1792 cells that carry KRAS 12C; and (**C**) CRISPR–Cas12a with crRNA targeting KRAS 12D detect the mutation only in the DNA of PANC-1 cells that carry KRAS 12D. (* *p* < 0.05).

Mutant KRAS DNA of the two cancer cells and wild-type KRAS DNA of BEAS2B cells were diluted in the genomic DNA isolated from the PBMCs of a healthy donor, respectively. The samples were tested by CRISPR–Cas12a and PCR analysis for the KRAS mutations. As shown in Figure 4, CRISPR–Cas12a and PCR unambiguously identified G12C, G12S, or G12D mutation, respectively. The results generated from the cells were in good agreement with those produced in plasmid DNA samples with the mutations (Figure 2).

Therefore, CRISPR–Cas12a could serve as a specific method to identify single-base mutation in DNA strands with the merits of excellent discrimination capacity for different RAS mutations.

### 3.4. CRISPR–Cas12a Can Sensitively Detect KRAS Mutations in the Tumor Tissues of Lung Cancer Patients

**Figure 5:**
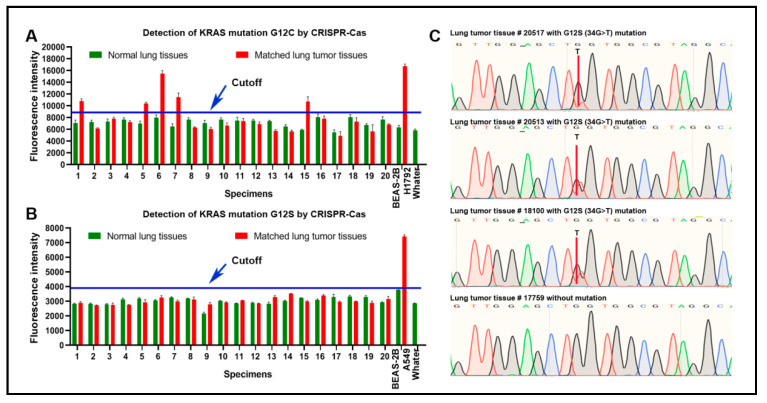
The sensitivity of CRISPR–Cas12a in comparison with direct DNA sequencing analysis of 20 pairs of lung tumor and normal lung tissues: (**A**) CRISPR–Cas12a could detect G12C mutation in five of 20 lung tumor tissues. H1792 cells carrying the G12C mutation were used as a positive control. BEAS-2B cells were used as a negative control; (**B**) there was no G12S mutation by CRISPR–Cas12a in the lung tumor tissues. A549 cells carrying G12S were used as a positive control. BEAS-2B cells were used as a negative control; (**C**) representative tumor tissue specimens (#20517, 20513, and 18100) that were positive for G12C mutation by CRISPR–Cas12a were also positive by DNA sequencing. A representative tumor tissue sample (#17759) that was positive for G12C mutation by CRISPR–Cas12a was negative by DNA sequencing.

This study aimed to develop CRISPR–Cas12a as a test to sensitively detect KRAS mutations in lung cancer. KRAS 12C and KRAS 12S mutations were more commonly observed in lung cancer, whereas G12D mutation was frequently found in pancreatic cancer. Therefore, we examined the presence of KRAS 12C and KRAS 12S mutations in 20 lung tumor tissues and the matched noncancerous tissues by using CRISPR–Cas12a, PCR, and direct sequencing. The three different platforms were successfully performed in the surgically resected tissue specimens. The mean and standard deviation (SD) of fluorescence signals of CRISPR–Cas12a in all normal lung tissue specimens were 7257 and 742 for G12C, and 3050 and 306 for G12S, respectively. We set a cut-off value for determining positive mutation by adding two SDs on top of the mean of fluorescence signals obtained from the normal lung tissue specimens. As a result, the cutoff values for positive G12C mutation and G12S mutation were 8742 and 3663, respectively (Figure 5A,B). Based on the cutoff values, CRISPR–Cas12a identified G12C mutation in five (25%) of the 20 tumors, an incidence of the mutation in lung cancer consistent with previous studies [15,16]. Both PCR and direct sequencing could detect the KRAS G12C mutation in three tumor tissues. The five tumor tissues that were positive for KRAS G12C mutation by CRISPR–Cas12a included the three specimens that were positive by both PCR and direct sequencing (Figure 5C). The two tumor tissues that was positive for KRAS G12C mutation by CRISPR–Cas12a was negative by PCR and direct sequencing analysis. Therefore, Cas12a had a higher sensitivity (25%) compared with both PCR and direct sequencing analyses (15%) for the detection of KRAS mutation (All *p* < 0.01) in clinical tissue specimens.

### 3.5. The Results of CRISPR–Cas12a for Testing KRAS Mutations Can Be Immediately Read by a UV Light Illuminator with Naked Eyes

**Figure 6:**
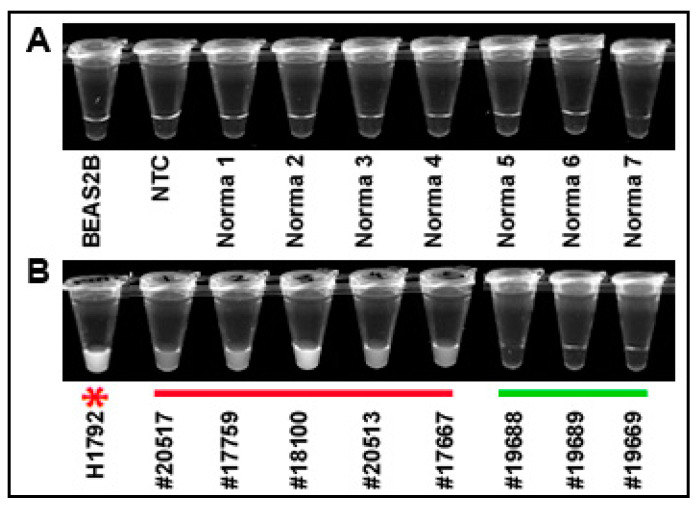
Directly visualizing the results of CRISPR–Cas12a for the detection of G12C mutation by naked eyes: (**A**) BEAS2B normal lung cells, NTC, and seven representative noncancerous lung tissue specimens had a negative result; (**B**) the five lung tumor specimens underlined in red and the H1792 cancer cells with a red star (#20517, 17759, 20513, 17567, and 18100) were positive for the 12G12 mutation as demonstrated by super-bright fluorescence signals. Three representative lung tumor tissue specimens underlined in green (#19688, 19689, and 19669) were negative for the mutation as demonstrated by the lack of fluorescence signal.

To determine if the results of CRISPR–Cas12a could be simply detected, the solution of the CRISPR reaction in micro-centrifuge tubes was observed by a UV light illuminator. CRISPR–Cas12a reaction in BEAS2B cells, no template control (NTC), and normal lung tissues did not exhibit fluorescence (Figure 6A). The super-bright fluorescence signal of the positive results for KRAS G12C mutation could be directly visualized as early as 10 min in H1792 cells and the five lung tumor tissues (#20517, 17759, 20513, 17567, and 18100) (Figure 6B). However, no fluorescence signal was observed in the three lung tumor tissue specimens that had no KRAS mutation (Figure 6B).

## 4. Discussion

The detection of KRAS mutations could help diagnose primary tumors at the early stage and guide treatment decision [17]. Given a low concentration of mutant-type KRAS DNA in a large excess of wild-type DNA in clinical specimens, the detection of a KRAS mutation requires a very sensitive assay in laboratory settings. However, the current techniques for KRAS mutation detection have unsatisfactory sensitivity [4,18]. The technique that can increase sensitivity for the detection of KARS mutations is urgently needed. CRISPR–Cas biology has revolutionized the field of molecular diagnostics for various diseases, since it has an extremely high sensitivity for the detection of nucleic acids [8]. In this study, we developed CRISPR–Cas12a as a novel assay that could sensitively and specifically detect KRAS mutations. The detectable mutant alleles by CRISPR–Cas12a were down to 0.01%, whereas this was 0.1% by PCR in serially diluted DNA samples containing KRAS mutations. Furthermore, the developed CRISPR–Cas12a strategy was successfully applied for the detection of KRAS mutation in tissue samples of lung cancer patients. Interestingly, of the five lung tumor tissues that were tested positive by CRISPR–Cas12a, only three were positive by both PCR and DNA sequencing analyses. Therefore, CRISPR–Cas12a might have a higher sensitivity for mutation detection in the clinical tissue specimens, compared with the conventional platforms. Of the clinical tissue specimens that were positive for KRAS mutations, all had the G12C (34G > T) mutation. The results are consistent with the previous findings that G12C is the most common mutation, while G12S only accounts for less than 1% of the KRAS mutations in lung cancer [3,15,16]. In addition, given the urgent need to identify the mutations of an increasing number of other genes (e.g., KIT, EGFR, BRAF, and PI3KCA) in many different types of human tumors, adapting the CRISPR–Cas12a technique for testing other genes would also have important applications in the clinical settings.

Although the results of this study look promising, the sample size of clinical specimens is small. A sufficiently large sample size is needed to prospectively validate the clinical significance of CRISPR–Cas12a for the detection of KRAS mutations. Furthermore, the analysis of plasma circulating tumor DNA (ctDNA) for KRAS mutations can help the real-time clinical management of patients when surgically resected tissues and re-biopsies are not available [19,20,21,22,23]. In addition, KRAS mutations are also common in head and neck cancer and sarcoma [24]. This new assay for the detection of KRAS mutation would have an important application for the diagnosis of other types of tumors, such as head and neck cancer and sarcoma. Our ongoing study was investigating if CRISPR–Cas12a could be used for sensitively detecting KRAS mutations in plasma ctDNA samples.

## 5. Conclusions

We demonstrated that CRISPR–Cas12a could achieve a superior sensitivity for the detection of KRAS mutations. Nevertheless, validating the novel assay in large sample studies is required.

## Figures and Tables

**Table 1:** Sequences of crRNA and reporter substrates.

crRNA’s Names	Sequence (5′->3′)
LbaCas12a crRNA-34G	AGCUGGUGGCGUAGGCA
LbaCas12a crRNA-G12C-34T	AGCUUGUGGCGUAGGCA
LbaCas12a crRNA-G12S-34A	AGCUAGUGGCGUAGGCA
LbaCas12a crRNA-G12D-35T	AGCUGUUGGCGUAGGCA
Hairpin	UAAUUUCUACUAAGUGUAGAU
Substrate’s name	Sequence (5′->3′)
ssDNA-FQ reporter	/56-FAM/TTATT/3IABKFQ/

## Supplementary Materials

The following are available online at https://www.mdpi.com/2075-4418/11/1/125/s1
