# Supplementary material for: Sensitive Detection of KRAS Mutations by Clustered Regularly Interspaced Short Palindromic Repeats
Source: Diagnostics (Basel). 2021 Jan 15;11(1):125. doi: 10.3390/diagnostics11010125 (PMC7830957; doi:10.3390/diagnostics11010125)
Supplement: Supplementary file 1 [file diagnostics-11-00125-s001.pdf]

Supplementary file

Supplemental table 1. Primer of KRAS of PCR for CRSIPR

Primer name	Sequence (5'->3')
Kras-F	AACTTGTGGTAGTTTGAGCT
Kras-R	TCTATTGTTGGATCATATTC

Supplemental table 2. Primers of KRAS of PCR for DNA sequencing analysis

Primer name	Sequence (5'->3')
EX2-F	5′-AACTTGTGGTAGTTGGAGCTG-3′
EX2-R	5′-GTTGGATCATATTCGTCCACA-3′
